# Topological data analysis (TDA) enhances bispectral EEG (BSEEG) algorithm for detection of delirium

**DOI:** 10.1038/s41598-020-79391-y

**Published:** 2021-01-11

**Authors:** Takehiko Yamanashi, Mari Kajitani, Masaaki Iwata, Kaitlyn J. Crutchley, Pedro Marra, Johnny R. Malicoat, Jessica C. Williams, Lydia R. Leyden, Hailey Long, Duachee Lo, Cassidy J. Schacher, Kazuaki Hiraoka, Tomoyuki Tsunoda, Ken Kobayashi, Yoshiaki Ikai, Koichi Kaneko, Yuhei Umeda, Yoshimasa Kadooka, Gen Shinozaki

**Affiliations:** 1 Department of Psychiatry, University of Iowa Carver College of Medicine, Iowa City, IA USA; 2 Department of Neuropsychiatry, Faculty of Medicine, Tottori University, Yonago, Japan; 3 FUJITSU LABORATORIES LTD, Tokyo, Japan; 4 FUJITSU LTD, Tokyo, Japan; 5 Department of Neurosurgery, University of Iowa Carver College of Medicine, Iowa City, IA USA; 6 Department of Anesthesia, University of Iowa Carver College of Medicine, Iowa City, IA USA; 7 Iowa Neuroscience Institute, Iowa City, IA USA; 8 Interdisciplinary Graduate Program in Neuroscience, University of Iowa, 25 S Grand Ave. Medical Laboratories B002, Iowa City, IA 52246 USA

**Keywords:** Computational neuroscience, Diagnostic markers

## Abstract

Current methods for screening and detecting delirium are not practical in clinical settings. We previously showed that a simplified EEG with bispectral electroencephalography (BSEEG) algorithm can detect delirium in elderly inpatients. In this study, we performed a post-hoc BSEEG data analysis using larger sample size and performed topological data analysis to improve the BSEEG method. Data from 274 subjects included in the previous study were analyzed as a 1st cohort. Subjects were enrolled at the University of Iowa Hospitals and Clinics (UIHC) between January 30, 2016, and October 30, 2017. A second cohort with 265 subjects was recruited between January 16, 2019, and August 19, 2019. The BSEEG score was calculated as a power ratio between low frequency to high frequency using our newly developed algorithm. Additionally, Topological data analysis (TDA) score was calculated by applying TDA to our EEG data. The BSEEG score and TDA score were compared between those patients with delirium and without delirium. Among the 274 subjects from the first cohort, 102 were categorized as delirious. Among the 206 subjects from the second cohort, 42 were categorized as delirious. The areas under the curve (AUCs) based on BSEEG score were 0.72 (1st cohort, Fp1-A1), 0.76 (1st cohort, Fp2-A2), and 0.67 (2nd cohort). AUCs from TDA were much higher at 0.82 (1st cohort, Fp1-A1), 0.84 (1st cohort, Fp2-A2), and 0.78 (2nd cohort). When sensitivity was set to be 0.80, the TDA drastically improved specificity to 0.66 (1st cohort, Fp1-A1), 0.72 (1st cohort, Fp2-A2), and 0.62 (2nd cohort), compared to 0.48 (1st cohort, Fp1-A1), 0.54 (1st cohort, Fp2-A2), and 0.46 (2nd cohort) with BSEEG. BSEEG has the potential to detect delirium, and TDA is helpful to improve the performance.

## Introduction

Delirium among elderly inpatients is very common, expensive, and dangerous^1–3^. Delirium is also difficult to be diagnosed and therefore less likely to be treated^4–6^. Delirium is prevalent in older adult inpatients, occurring in up to 50% of patients admitted to general internal medicine floors, 15–53% who are undergoing post-operative recovery, and 70–87% who are in intensive care units (ICU)^1,7^, which translates to a minimum of around three million cases of delirium annually in the U.S. alone. Delirium is especially common among elderly patients with dementia, and it strongly predicts poor patient outcomes, such as increased rates of mortality^4,8–11^, length of stay in the hospital, and institutionalization after discharge^1–3^. Even when these patients survive, their risk of long-term cognitive impairment is high^12^ and progression of baseline dementia is accelerated^2,13^. If undetected, delirium can cost more than $60,000 per patient every year, i.e., > $150 billion in added healthcare costs in the U.S. alone^3,5^. The burden for families taking care of patients with delirium is also significant and can be traumatic^14^. Thus, undiagnosed or untreated delirium has widespread effects on individual patients and the healthcare system as a whole.

It has been shown that low-technology interventions can prevent occurrence of delirium cases^3,15–17^, and in 1990 the Confusion Assessment Method (CAM) was introduced as a method for delirium detection^18^. This led to extensive efforts to identify useful tools for screening and detecting delirium using various questionnaire-style instruments, including the Confusion Assessment Method for Intensive Care Unit (CAM-ICU)^19,20^ and the Delirium Rating Scale-Revised-98 (DRS)^21^. Despite these instruments being effective when rigorously implemented, delirium remains seriously underdiagnosed and undertreated^4–6^, in part because these methods are often challenging to apply in a hospital setting because they involve extensive questionnaires administered multiple times daily by busy hospital personnel. Also, their subjective nature makes it difficult to monitor change over the course of a hospital stay, particularly when they are used by different staff members. Because of the challenges, it has been shown that these tools have suboptimal sensitivity (38–47%) when used in busy clinical settings including the ICU^22,23^. Another important limitation is that these approaches detect delirium only after it has developed. These situations make it clear there is a critical need for more objective and effective detection of delirium followed by timely intervention in order to reduce the healthcare costs related to the increased morbidity and mortality in patients with this disorder.

Electroencephalography (EEG) is useful for detecting brain wave signals that are characteristic of delirium^24–28^. However, there are several obstacles that prevent its regular utilization for screening of many hospitalized patients. First, a traditional standard multiple-lead EEG instrument is usually heavy, large and not portable; it is cumbersome to transport and expensive to purchase, thus access to such machines is limited for most patients who may not exhibit obvious indication for EEG. Second, a well-trained technician must work for a significant amount of time to correctly position the numerous EEG electrodes on a patient’s scalp, and a neurologist specialized in electrophysiology must interpret the data and report the findings in the record. This results in significant delay in starting treatment for patients with abnormal brain signals suggestive of delirium. These limitations associated with the current resources have prevented widespread adoption of traditional EEG use for mass screening of delirium, even though the use of such analysis could improve detection and diagnosis, ultimately lowering the quality and cost of care.

Electrophysiological signals that are characteristic of delirium are commonly described as “*diffuse slowing”* in traditional EEG recordings. The term *diffuse slowing* indicates that in individuals experiencing delirium, the activity across most or all of the 20 electrodes (diffuse) is of low frequency (slowing). The emergence of low-frequency waves is an indication that a patient has global brain dysfunction commonly seen in delirium. The fact that all 20 leads detect the same slowness in frequency indicates that fewer leads are sufficient to obtain the relevant data. Thus, we developed a novel bispectral EEG (BSEEG) system that utilizes only two EEG channels, and these can be easily applied by non-experts. In addition, no special expertise is needed for interpretation of the data with appropriate signal processing. We conducted a study using this novel BSEEG method for the detection of slowing brain waves that are characteristic of delirium. Our published study showed that the BSEEG method is useful in detecting delirium among elderly inpatients^29,30^. Although our published data on BSEEG and other literature about EEG support the notion that such approaches are useful for detecting delirium^29–32^, challenges existed in accuracy of performance, and no large-scale study has been conducted to see whether improvement of performance through advanced signal analysis technology in detecting delirium using the BSEEG method is possible.

Topological data analysis (TDA) provides a relatively new, emergent general framework for the analysis of data that has the advantages of being able to capture global information from complex and large volume of data and to provide stability against noise. TDA uses mathematical approaches in algebraic topology to provide quantitative information for the analysis of the "shape" of data^33^. A prominent branch of TDA is persistent homology, which analyzes the dynamics of the topological features of a data set in the form of holes. The basic idea of persistent homology is to construct a figure that changes according to the parameters of the real number of parameters from the point cloud data sampled in the Euclidean space, and then extract information about the original point cloud in the form of changes in the homology of the figure according to the changes in the parameters, that is, changes in the connected components and the number of holes^33^. For time series data including EEG, it extracts global features such as waveform dynamics. In recent years Topological Persistent homology has been successfully applied to a range of applications, for example, image^34^, neuron^35^, action^36^, aircraft^37^, heart-beat data^38^, and so on.

In the present study, we aimed to determine whether detection of delirium among elderly patients using the BSEEG method can be improved with advanced signal analysis technology, especially TDA. To validate our findings, we used two independent cohorts with two different EEG devices.

## Results

### Participant demographics

**Figure 1:**
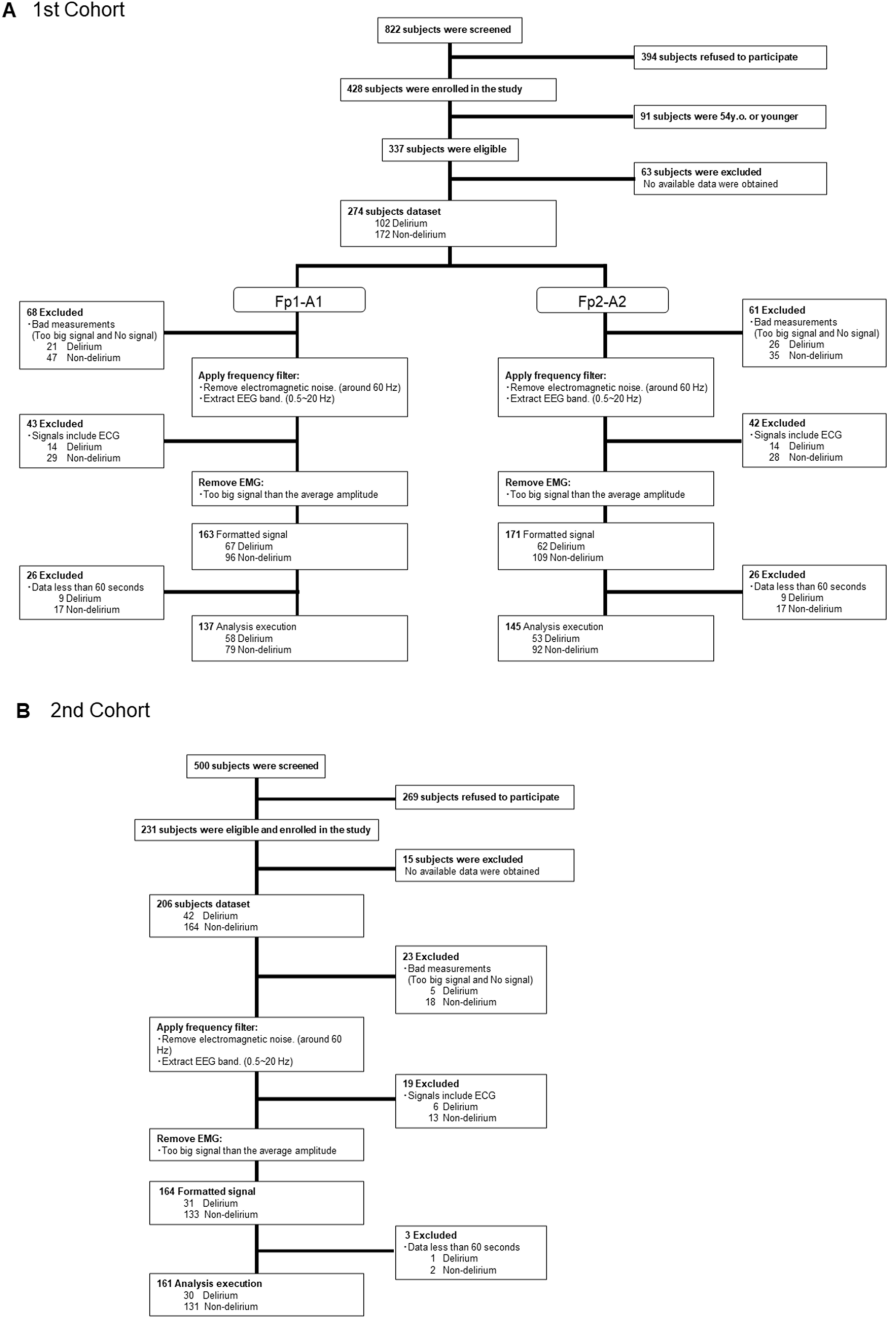
Flow of participants through the study. (**A**) cohort 1, (**B**) cohort 2.

The average patient age from the first cohort was 73.5 yo (SD = 9.6), 53.6% of subjects were female, and 98.9% were non-Hispanic white (NHW) per self report. In the 1st cohort, among the 274 subjects, 102 were categorized as delirious, and 172 were judged not to have delirium at the time of assessment (Table 1). The average patient age from the second cohort was 70.9 yo (SD = 9.8), 46.6% of subjects were female, and 92.7% were non-Hispanic white (NHW) per self report. In the 2nd cohort, among the 206 subjects, 42 were categorized as delirious, and 164 were judged not to have delirium at the time of assessment (Table 1). Age, sex, or race were not significantly different between the delirious and control groups for both cohorts except age for the 2nd cohort. DRS, the Delirium Observation Screening Scale (DOSS) and the Montreal Cognitive Assessment (MoCA) were significantly different between groups for both cohorts. Charlson Commobidity Index (CCI) for 1st cohort, but not 2nd cohort, was significantly higher in the delirious group than in control group. The Clinical Dementia Rating (CDR) score for the 2nd cohort was significantly higher in the delirious group than in the control group (Table 1). Flow of participants through the study is shown in Fig. 1.

**Table 1:** Patient characteristics.

Classification	1st Cohort				2nd Cohort			
	Clinical categories		Statistical test	p-value	Clinical categories		Statistical test	p-value
	Delirious (N = 102)	Control (N = 172)			Delirious (N = 42)	Control (N = 164)		
Mean age—year	73.8	73.3	t = 0.34	0.37	78.0	69.1	t = − 5.60	< 0.01
SD	9.4	9.8			8.0	9.4		
Female sex (n)	52	95	χ^2^ = 0.47	0.50	17	79	χ^2^ = 0.80	0.37
%	51.0%	55.2%			40.5%	48.2%		
Race			χ^2^ = 0.65	0.42			χ^2^ = 1.67	0.20
White (n)	101	168			37	154		
%	99.0%	97.7%			88.1%	93.9%		
Other (n)	1	4			5	10		
%	1.0%	2.3%			11.9%	6.1%		
Mean CCI	4.2	3.4	t = 2.13	< 0.05	4.3	3.7	t = − 1.15	0.25
Mean DRS	16.9	6.6	t = 14.07	< 0.01	12.4	5.5	t = − 13.85	< 0.01
Mean DOSS	6.3	0.2	t = 10.41	< 0.01	5.7	0.7	t = − 10.04	< 0.01
Mean MoCA	14.4	23.4	t = − 9.36	< 0.01	11.5	22.8	t = 13.95	< 0.01
Mean CDR	NA	NA	NA	NA	0.63	0.28	t = − 4.78	< 0.01

*CCI* Charlson commobidity index, *DRS* delirium rating scale-revised-98, *DOSS* Delirium observation screening scale, *MoCA* Montreal Cognitive assessment, *CDR* clinical dementia rating.

### Signal pre-processing

**Figure 2:**
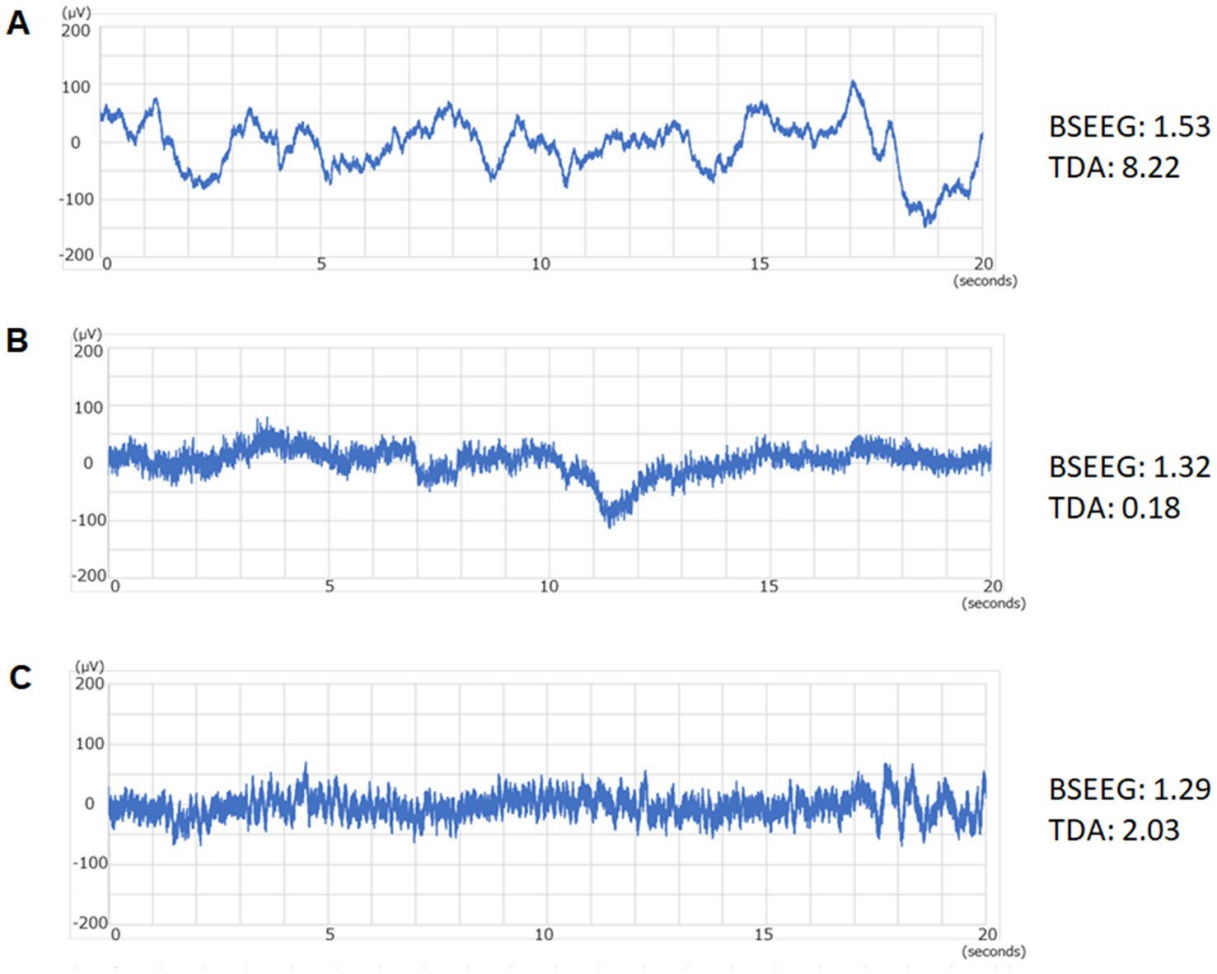
Examples of EEG signals in case of (**A**) obvious slowing where both BSEEG and TDA show positive scores, (**B**) obvious normal signals where both BSEEG and TDA show negative scores, and (**C**) not obvious slowing where BSEEG show negative scores, but TDA show positive scores.

**Figure 3:**
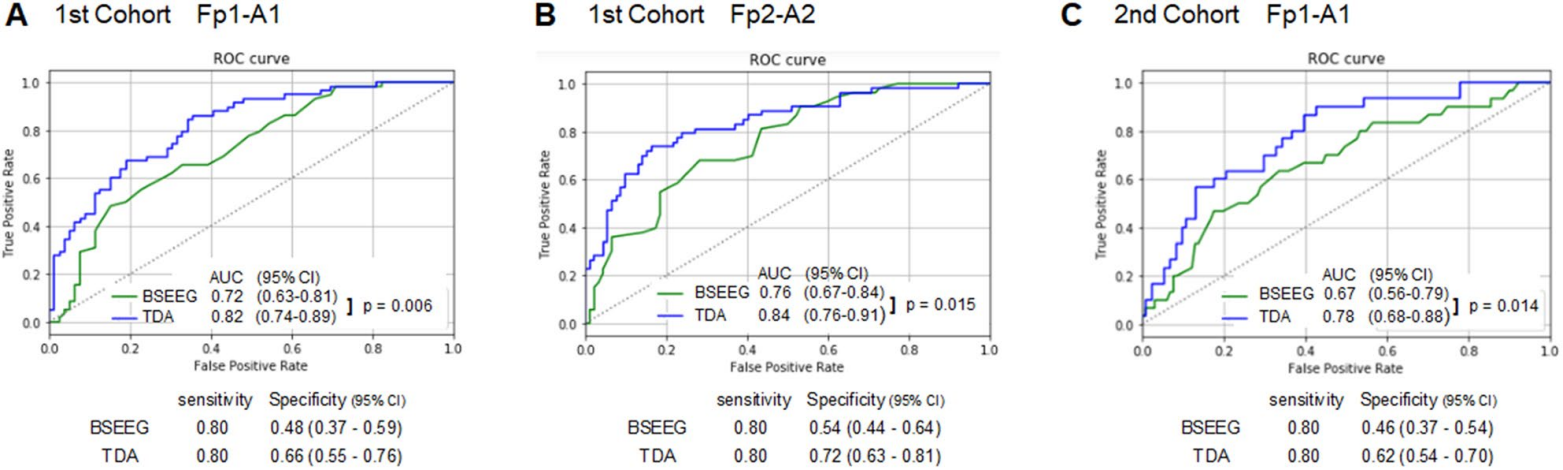
(**A**) ROC based on signals obtained from Fp1-A1 from the first cohort (58 delirium cases and 79 controls), (**B**) ROC based on signals obtained from Fp2-A2 from the first cohort (53 delirium cases and 92 controls), and (**C**) ROC based on signals obtained from Fp1-A1 from the second cohort (30 delirium cases and 131 controls). AUCs, DeLong's test results, and specificities when sensitivities were set for 0.80 are shown.

After pre-processing, around half of the cases remained suitable for analysis by TDA for both cohorts. Detailed numbers of each subjects from both channels (Fp1-A1 and Fp2-A2) from 1st cohort, as well as 2nd cohort are shown (Supplementary Table). Examples of EEG signals before and after pre-processing show significant reduction of artifacts from raw siganls (Supplementary Fig. S1). Also, representative EEG signals and their corresponding BSEEG scores, as well as TDA scores, are shown (Fig. 2). Examples include (1) delirium patient with slowing in brain wave and high (positive) scores with both BSEEG and TDA (Fig. 2A), (2) non-delirium control with clear normal brain wave and low (negative) scores with both BSEEG and TDA (Fig. 2B), and (3) delirium patient with unclear brain wave with low (negative) BSEEG score, but with high (positive) TDA score (Fig. 2C).

### Delirium detection performance

#### 1st Cohort

The BEEG scores, TDA scores, and delirium status categories are listed in the Supplementary Table. The area under the curve (AUC) from the receiver operating characteristic (ROC) curve analysis is shown in Fig. 3A,B for Fp1-A1 and Fp1-A2, respectively. AUCs based on BSEEG score obtained for day 1 after enrollment to the study were 0.72 (Fp1-A1: 95% CI 0.63–0.81) and 0.76 (Fp2-A2: 95% CI 0.67–0.84) . In contrast, AUCs from TDA were much higher at 0.82 (Fp1-A1: 95% CI 0.74–0.89) and 0.84 (Fp2-A2: 95% CI 0.76–0.91). AUCs from TDA were significantlly higher than AUCs from BSEEG (Fp1-A1: p = 0.006, Fp2-A2: p = 0.015). When sensitivity was set to be 0.80, TDA drastically improved specificity to 0.66 (Fp1-A1: 95% CI 0.55–0.76) and 0.72 (Fp2-A2: 95% CI 0.63–0.81) compared to 0.48 (Fp1-A1: 95% CI 0.37–0.59) and 0.54 (Fp2-A2: 95% CI 0.44–0.64)) with BSEEG (Fig. 3A,B). BSEEG and TDA result distributions are shown in Supplementary Fig. S2.

#### 2nd Cohort

The BEEG scores, TDA scores, and delirium status categories are listed in the Supplementary Table. The AUC from the ROC curve analysis is shown in Fig. 3C. AUCs based on BSEEG score obtained for day 1 after enrollment in the study were 0.67 (95% CI 0.56–0.79). In contrast, AUCs from TDA were much higher at 0.78 (95% CI 0.68–0.88). AUC from TDA were significantlly higher than AUC from BSEEG (p = 0.014). When sensitivity was set to be 0.80, TDA drastically improved specificity to 0.62 (95% CI 0.54–0.70) compared to 0.46 (95% CI 0.37–0.54) with BSEEG (Fig. 3C). BSEEG and TDA result distributions are shown in Supplementary Fig. S2.

### Adverse events

No adverse events from performing EEG data collection or evaluating clinical data were reported.

## Discussion

This is the first study to apply TDA for EEG signals obtained by BSEEG to detect delirium. BSEEG methods were developed to bring the merit of early detection of delirium to clinical practice. TDA was shown to improve the detection accuracy of BSEEG methods, further making this approach more reliable. The results were replicated with two independent cohorts, by two different portable EEG devices. As shown in Fig. 2, there are several situations observed with EEG recordings. In typical slowing waves obvious to the human eye (Fig. 2A), both BSEEG and TDA correctly identified those with high scores in delirium patients. In normal brain waves, both BSEEG and TDA correctly analyzed those with low scores in non-delirium controls (Fig. 2B). The benefit of TDA over BSEEG was also shown in the case of EEG signals not clear with slowing and BSEEG showed low score, altough TDA correctly calculated high score to capture delirium case (Fig. 2C). Although BSEEG was simple and effective to capture most of the slow EEG waves from this point-of-care device, TDA showed superior performance in capturing challenging cases to improve better accuracy.

The merit of BSEEG and TDA is its objectiveness compared to currently available questionare-style screening instruments. Also, this method does not require a large EEG machine, which is not suitable for large-volume mass screening in the hospital. A specialized technician for multiple electrode placement is not necessary, and expert interpretation is not required. It is easy to use by busy hospital staff, with minimal training and interpretation required. However, the challenge of the previous algorithm was the relatively limited accuracy for detection of delirium in the range of AUC of 0.67–0.76^29^. However in this report, we show that an additional signal processing analysis method, TDA, can overcome the challenge of limited accuracy and enhance the reliability of the BSEEG approach by increasing specificity by 16%-18%.

The current analysis has challenges and limitations. First, obtained raw EEG data are inevitably contaminated with multiple sources of potential artifacts including EMG (Supplementary Fig. S1). It has been reported that since EEG frequency bands and EMG frequency bands overlap it is difficult to remove EMG from EEG by band pass filter alone^39^. We need to improve EEG device hardware and to advance the algorithmic approach to overcome this problem. However, our previous and present data showed that BSEEG or TDA score analyzed from the potable devices have a promising potential to be useful to detect delirium and to predict poor outcomes, even with the limitation mentioned above. Second, as described above, pre-processing to select EEG signals suitable for TDA limited available samples to 60 ~ 80% of original samples. Part of this drastic decrease is due to our rigorous criteria for acceptance of EEG signals to verify that they are suitable for TDA. However, such a stringent approach excluded many samples for the analysis, which is not optimal for future application in clinical practice. To overcome this challenge, we are actively working towards finding a good balance between restricting poor-quality signals versus including them with improved signal processing (both pre- and post-filtering). Another challenge is that for this TDA analysis, several parameters were adjusted to achieve the best performance. It is not yet proven that those specific parameters would be the best for data from additional independent samples. For example, with our two independent cohorts with two different EEG devices, TDA parameters needed to be fine tuned to show similar improvement of detection performance. However, we tested the same TDA analysis parameters for two different datasets from Fp1-A1 and Fp2-A2 and both data showed consistent benefit. Another limitation is that this data is from a single institution in the Midwest region of the U.S, and generalizability needs to be tested in different institutions with more diverse ethnic backgrounds, as our study participants were mainly NHW. Lastly, TDA is a computationally intensive algorithm at this point. Therefore, we obtained EEG data at the bedside, and then we analyze EEG data using a PC in the laboratory. Thus, we could not tell whether we could obtain TDA data at the bedside. We should be able to avoid missing data if we can analyze EEG data at the bedside, as we can potentially repeat measurements until we obtain good quality signals suitable for TDA analysis. Currently we are working to enhance the speed of the algorithm so that it can be implemented into a handheld device; we envision this approach will be used at the bedside. Although there are several limitations, our data presented here showed the promise of TDA applied to BSEEG to enhance more accurate and objective detection of delirium with this novel approach.

Once early detection of brain dysfunction associated with poor outcomes such as mortality is made, it is possible to identify reversible causes, followed by early intervention and close monitoring to avoid preventable complications. Such an approach may shorten hospital stay, increase a patient’s chance to go home, decrease mortality, and suppress financial loss for hospitals.

## Conclusion

In summary, delirium is a dangerous condition and early detection is vital for better outcomes, but current methods are suboptimal and not practical. We showed that a simplified EEG with BSEEG algorithm has the potential to detect delirium, and TDA is helpful to drastically improve the performance. We validated our results using two independent cohorts with two different portable EEG devices. This approach is easy to use in busy hospital settings and would potentially benefit patients, physicians, hospitals, and the economy of health care.

## Methods

### Study design

This is a post-hoc analysis of data obtained through a prospective, cohort study invesitgating the role of BSEEG to detect delirium and predict patient outcomes^40^. We also used a second independent cohort for replication using a different EEG device. The protocol of this study was approved by the University of Iowa (UI) Institutional Review Board. This study conforms to the provisions of the Declaration of Helsinki.

### Setting and participants

For this report, first, we analyzed data from 274 subjects included in the previous study enrolled at the UI Hospitals and Clinics (UIHC) between January 30, 2016, and October 30, 2017 (1st cohort)^40^. Second, we collected and analyzed data from an additional 206 subjects recruited at UIHC between January 16, 2019, and August 19, 2019 (2nd sohort). A detailed description of the recruitment process has been reported previously^40^. Eligibility criteria was 1) subjects who were admitted to the general medicine floor, the orthopedics floor, or the emergency department; 2) subjects whose age were 55-years or more. Potentially eligible subjects were identified through reviewing the electronic medical record.

We assessed the eligibility of patients for their capacity to consent and participate. When the subjects displayed the capacity to provide consent, they consented on their own. If the subject was delirious and it was determined they did not have the capacity to consent, their legally authorized representative signed the consent on their behalf. We obtained written informed consent from participants or their legally authorized representative after providing a complete description of the study. Study subjects were recruited from a convenience series of eligible patients.

### Clinical data collection and case definition

Details of clinical data collection methods and case definitions have been described previously^40^. Briefly, medical history, as well as demographic characteristics and CDR score, were obtained from chart review and interviews. CCI was calculated based on subject’s medical record^41^. CAM-ICU^19,20^, DRS^21^, DOSS^42^ and MoCA^43^ were administered for clinical assessment. We defined a case of delirium by a positive score, or beyond the cut-off score on any of the questionnaires (CAM-ICU positive, DRS ≥ 19, or DOSS ≥ 3) or clinical documentation of evidence of delirium at the time of initial assessment as previously described^40^. A psychiatrist board-certified in consultation-liaison psychiatry (G.S.) reviewed each case in question for final determination for case definition. The clinical raters were blind for the EEG scoring as the EEG data was analyzed after clinical evaluation later in the laboratory.

### BSEEG data collection and score calculation

We obtained EEG data on the day of recruitment. In our previous study, a portable EEG device (CMS2100, CONTEC, Qinhuangdao, Hebei, China) was utilized to collect brain signals. Sampling rate of the EEG is 500 Hz. For our second cohort, a different EEG device (ZA, ProAssist, Osaka, Japan) was used and the sampling rate of this device was 128 Hz. We applied our newly developed algorithm of BSEEG from EEG signals obtained from the forehead of the study participants^29,30,40,44^. Signals were obtained from Fp1-A1 and Fp2-A2 from the 1st cohort. Signals were obtained from Fp1-A1 only from the 2nd cohort. The BSEEG score was calculated as a power spectral density (PSD) ratio between low frequency (3 Hz) to high frequency (10 Hz) as described previously^29,40,44^. In other words, PSD of low frequency (3 Hz) divided by PSD of high frequency (10 Hz) was BSEEG score. In addition to BSEEG score, this time we introduced TDA to enhance our algorithm. To apply TDA to the raw signals, rigorous pre-processing methods were employed. Raw signals were processed by low-pass and high-pass band filtering to capture signals between 0.5 Hz and 20 Hz, as well as removal of noises and artifacts such as signals from the echocardiogram. Also, we included signals for analysis if they contained EEG signals suitable for analysis over more than 30 s. The EEG data analyzers were blind for clinical data.

### TDA scoring

TDA score was calculated as the evaluation criteria for detecting delirium using TDA. TDA is a powerful tool for analyzing complex datasets. Our TDA processing pipeline applies persistent homology to time-delay embeddings that capture the underlying system dynamics from which time series data is acquired. TDA score was calculated as the area of a 1-dimensional Betti curve, one of the outputs from persistent homology, that represents irregularity of the time series. A full description of data processing is described in the supplementary material. Then we performed signal windowing, whereby we extracted each channel of data and subsequently divided each channel into 2-s windows and 4-s windows. Next, window filtering was performed, with each window of data being interrogated for excessive noise, and those windows with interference being removed from further analysis. Then we processed each remaining window for extraction of the following two signal features. First is the power spectral density ratio (PSDR) obtained via fast Fourier transformation of remaining 4-s windows as BSEEG score. Second is the result applying TDA processing pipeline to 2-s windows as TDA score. More specific details of the calculation process for TDA are described in the Supplementary Methods.

### Statistical analysis

Sample size of the 1st cohort was same as our previous study^40^, and the one of the 2nd cohort was determined based on the 1st cohort to be consistent with the original cohort. Demographics were compared using unpaired t-test for continuous variables or chi-square test for categorical variables. We used ROC curves and AUCs to analyze the relationship between delirium-positive cases and BSEEG or TDA. We performed DeLong’s test to compare the AUC from BSEEG and the one from TDA^45^. P-values < 0.05 were considered statistically significant. Missing and indeterminate BSEEG and TDA results were removed from the analyses (Fig. 1).

## Supplementary Information

Supplementary Information 1.

Supplementary Information 2.

Supplementary Information 3.

Supplementary Information 4.

Supplementary Information 5.

## Data Availability

Raw data are shown in Supplementary Table.
