# Supplementary figures and images for: Topological data analysis (TDA) enhances bispectral EEG (BSEEG) algorithm for detection of delirium
Source: Sci Rep. 2021 Jan 11;11:304. doi: 10.1038/s41598-020-79391-y (PMC7801387; doi:10.1038/s41598-020-79391-y)

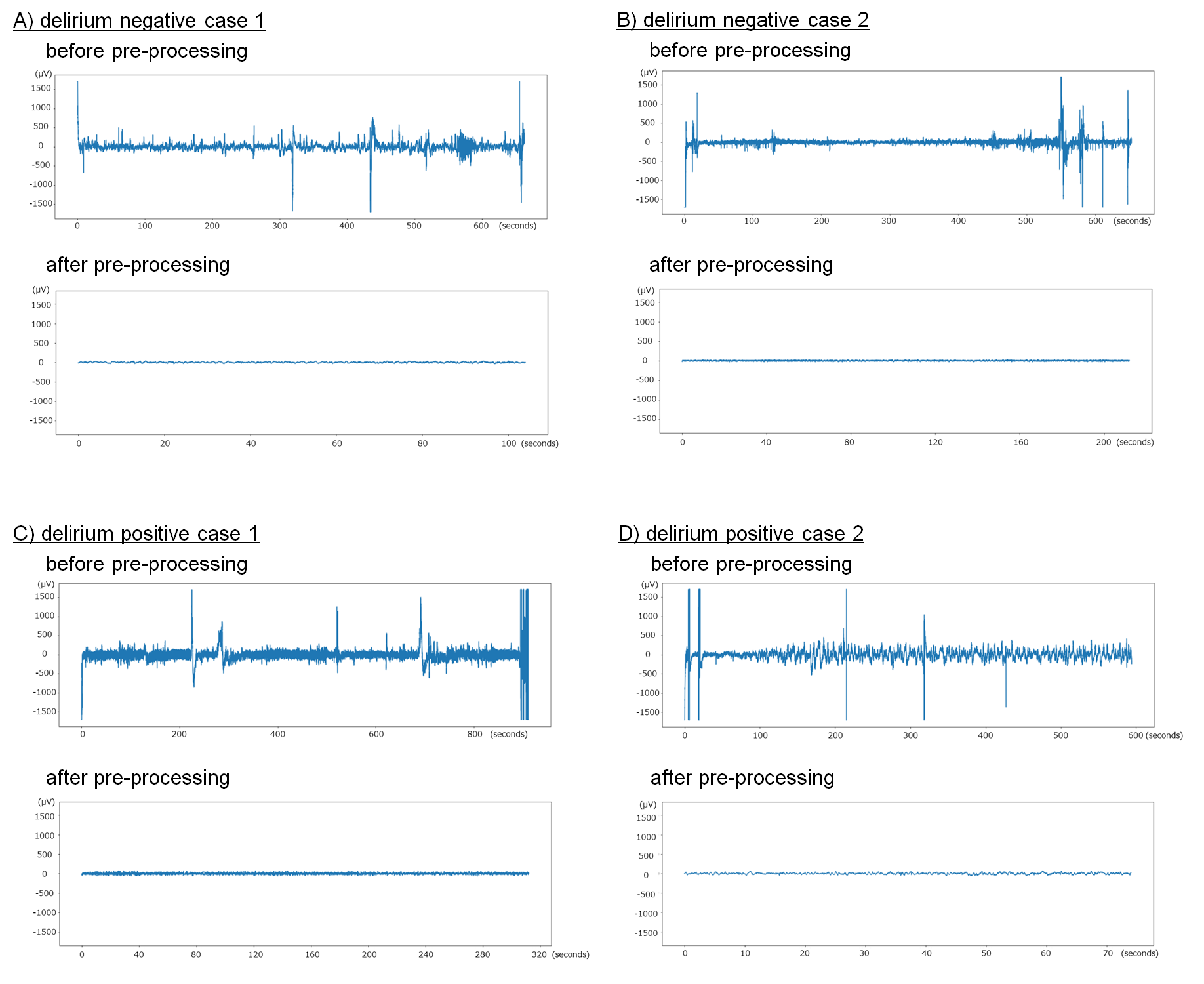

Supplement: Supplementary file 2 — Supplementary Information 2. [file 41598_2020_79391_MOESM2_ESM.tif]

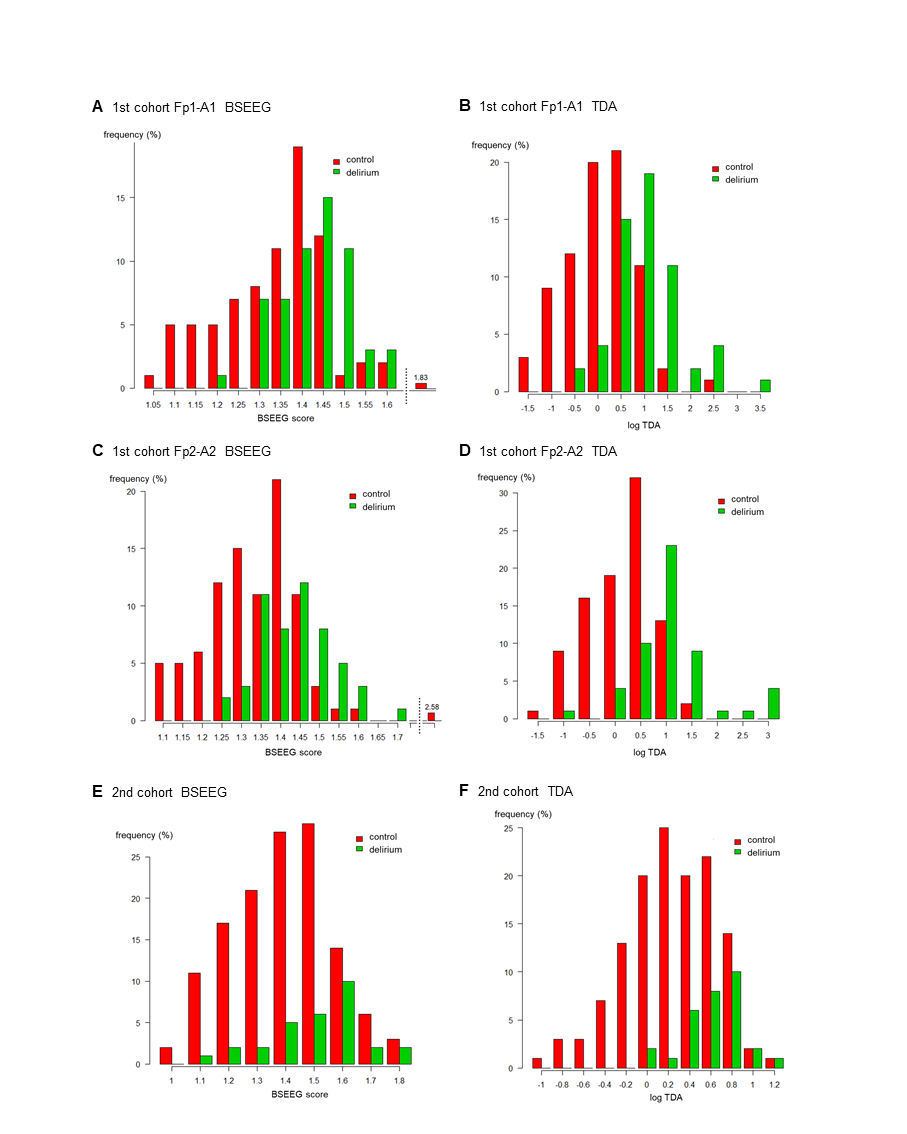

Supplement: Supplementary file 3 — Supplementary Information 3. [file 41598_2020_79391_MOESM3_ESM.tif]
