# Supplementary material for: Topological data analysis (TDA) enhances bispectral EEG (BSEEG) algorithm for detection of delirium
Source: Sci Rep. 2021 Jan 11;11:304. doi: 10.1038/s41598-020-79391-y (PMC7801387; doi:10.1038/s41598-020-79391-y)
Supplement: Supplementary file 4 — Supplementary Information 4. [file 41598_2020_79391_MOESM4_ESM.docx]

# Supplementary material

## Full description of processing of the TDA processing pipeline

### Noise removal

Because TDA capture the shape of waveform, we have to extract brain waves. So we apply bandpass filter and extract 0.5–—20 Hz elements.

### Time-delay embedding

In the 1st cohort, the 2-s windows have 1000 points x(1),x(2),...,x(1000) because the sampling rate of the EEG device used in this experiment is 500 Hz. We construct time-delay embedding in 3-dimensional Euclidean space from the window. Time-delay embedding is transformation from signal to point-cloud in Euclidean space represented as a set of sliding windows (x(1+kΔs), x(1+kΔs+Δt), x(1+kΔs+2Δt)), where k=1,2,…,m (see Fig.1). In this case, we set Δs = 3 and Δt = 24, that is, time delay embedding is represented as set of 316 points in 3-dimensional Euclidean space: (x(1),x(25),x(49)), (x(4),x(28),x(52)),(x(7),x(31),x(55)),...,(x(949),x(973),x(997)). This time delay embedding represents the underlying system dynamics from which time series data is acquired. This represent irregularity of time-series.

In the 2nd cohort, the 2-s windows have 256 points y(1),y(2),...,y(256) because the sampling rate of the EEG device used in this experiment is 128 Hz. In this case, we set Δs = 1 and Δt = 6, that is, time delay embedding is represented as set of 244 points in 3-dimensional Euclidean space: (y(1),y(7),y(13)), (y(2),y(8),y(14)),(y(3),y(9),y(15)),...,(y(244),y(250),y(256)).


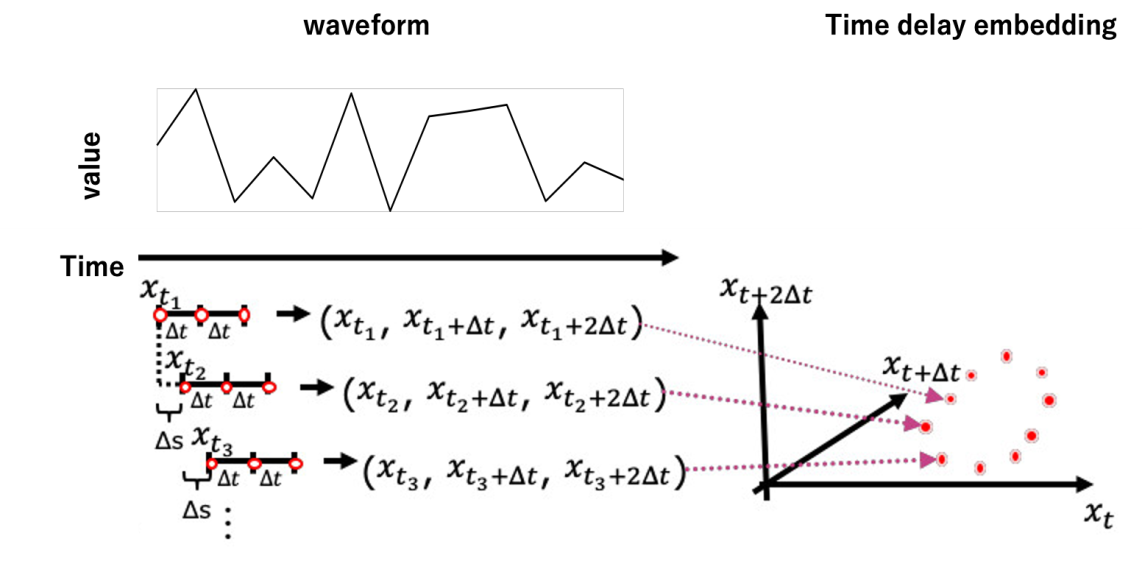


Fig. 1 Time-delay embedding

### 1-dimensional Betti curve

1-dimensional Betti number is the history of number of holes of union of balls centered at each points of time delay embedding as the radius of the ball increases. 1-dimensional Betti curve is a line graph that the vertical line shows 1-dimensional Betti number and the horizontal one radius of ball. Fig. 2 shows the history of union of balls as the radius increase and corresponding Betti curve.


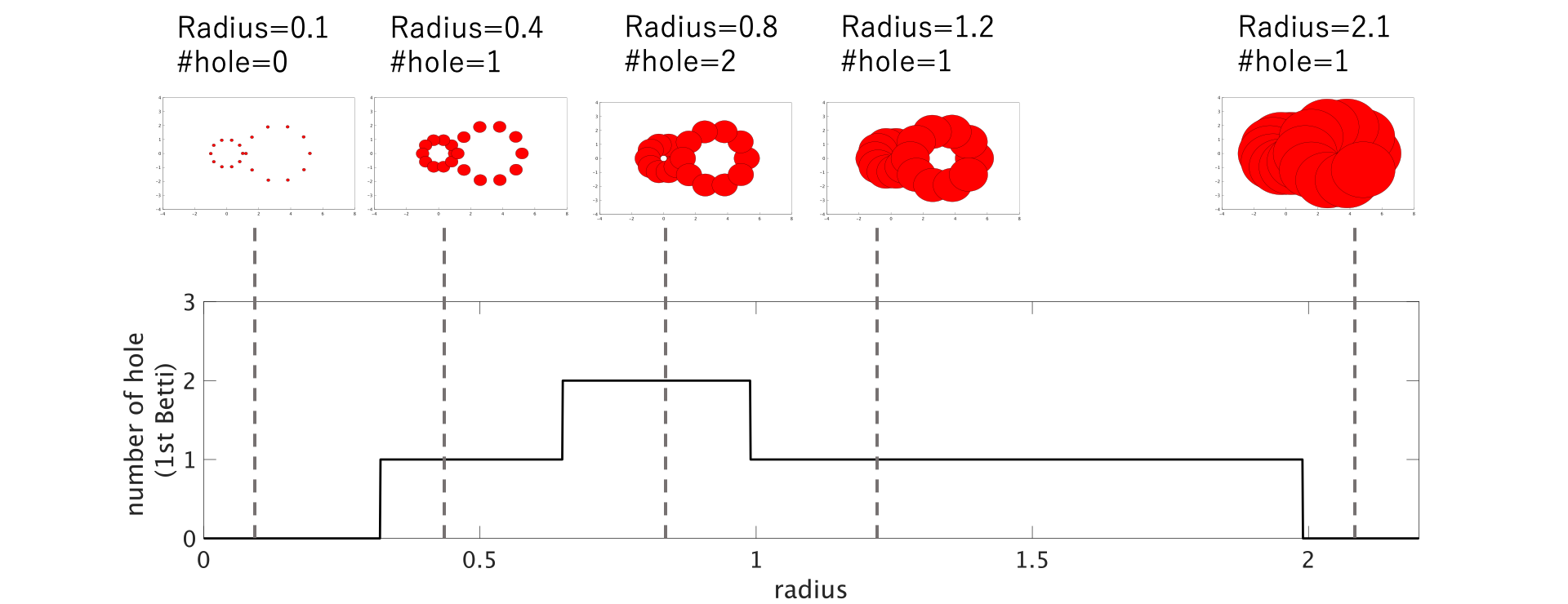


Fig. 2 Betti curve

### TDA feature

TDA feature was calculate as the area of 1-dimensional Betti curve.

### TDA processing pipeline

Each channel of 1-min data was extracted and each channel was subsequently divided into 2-s windows. Next, we performed window filtering, with removing artifact and ECG data. Each remaining window was then calculated TDA feature, and the mean value of them is used as the TDA score.

Fig. 3 shows examples of EEG waveforms and corresponding 1^st^ Betti curves for positive and negative cases, respectively. As can be seen from this figure, the area of the 1^st^ Betti curve tends to be larger in the positive case.


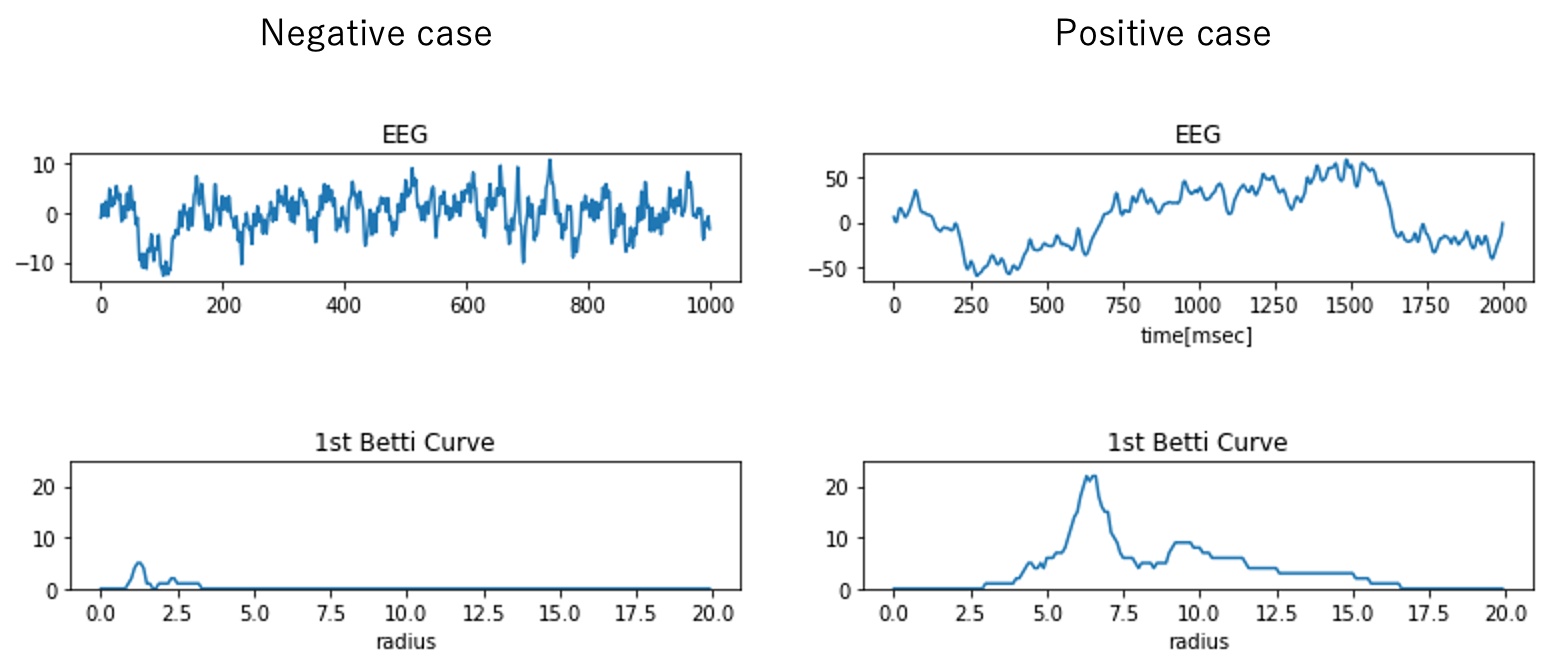


Fig. 3 EEG wave and corresponding 1^st^ Betti curve
