# Supplementary material for: Topological data analysis (TDA) enhances bispectral EEG (BSEEG) algorithm for detection of delirium
Source: Sci Rep. 2021 Jan 11;11:304. doi: 10.1038/s41598-020-79391-y (PMC7801387; doi:10.1038/s41598-020-79391-y)
Supplement: Supplementary file 5 — Supplementary Information 5. [file 41598_2020_79391_MOESM5_ESM.docx]

**Supplementary Figure 1:** Examples of EEG signals before and after pre-processing show significant reduction of artifacts from raw signals. A) 1st example of delirium negative case; B) 2nd example of delirium negative case; C) 1st example of delirium positive case; D) 2nd example of delirium positive case

**Supplementary Figure 2:** Distribution of BSEEG and TDA scores. A) cohort 1, Fp1-A1, BSEEG; B) cohort 1, Fp1-A1, TDA; C) cohort 1, Fp2-A2, BSEEG; D) cohort 1, Fp2-A2, TDA; E) cohort 2, BSEEG; F) cohort 2, TDA. TDA was shown using the logarithm.

**Supplementary Table:** Raw data of BSEEG score and TDA score from the 1st cohort Fp1-A1, 1st cohort Fp2-A2, and the 2nd cohort.
